# The Biological Reference Repository (BioR): a rapid and flexible system for genomics annotation

**DOI:** 10.1093/bioinformatics/btu137

**Published:** 2014-03-10

**Authors:** Jean-Pierre A. Kocher, Daniel J. Quest, Patrick Duffy, Michael A. Meiners, Raymond M. Moore, David Rider, Asif Hossain, Steven N. Hart, Valentin Dinu

**Affiliations:** ^1^Division of Biomedical Statistics and Informatics, Department of Health Sciences Research and ^2^Department of Research IT, Mayo Clinic, Rochester, MN 55905, USA and ^3^Department of Biomedical Informatics, Arizona State University, Scottsdale, AZ 85259, USA

## Abstract

**Motivation:** The Biological Reference Repository (BioR) is a toolkit for annotating variants. BioR stores public and user-specific annotation sources in indexed JSON-encoded flat files (catalogs). The BioR toolkit provides the functionality to combine and retrieve annotation from these catalogs via the command-line interface. Several catalogs from commonly used annotation sources and instructions for creating user-specific catalogs are provided. Commands from the toolkit can be combined with other UNIX commands for advanced annotation processing. We also provide instructions for the development of custom annotation pipelines.

**Availability and implementation:** The package is implemented in Java and makes use of external tools written in Java and Perl. The toolkit can be executed on Mac OS X 10.5 and above or any Linux distribution. The BioR application, quickstart, and user guide documents and many biological examples are available at http://bioinformaticstools.mayo.edu.

**Contact:** Kocher.JeanPierre@mayo.edu

**Supplementary information:** Supplementary data are available at *Bioinformatics* online.

## 1 INTRODUCTION

Next-generation sequencing (NGS) technology platforms are providing unprecedented opportunities to study genomic variants that are associated with clinical conditions and drug response. Using NGS technologies, researchers can identify mutations associated with rare diseases, characterize somatic variants in tumor for diagnostic or prognostic purpose or guide therapeutic treatment. Although the large amount of data produced by NGS platforms and the time to process them are largely being addressed by expanding the IT infrastructure, high-performance computing and code optimization, the annotation process needed to interpret the thousands of variants found in individual genomes is still a challenging task. The annotation process requires extracting and combining information from disparate external and in-house annotation sources, or even command-line tools. Several applications such as ANNOVAR (Wang *et al.*, 2010), GEMINI (Paila *et al.*, 2013) and TREAT (Asmann *et al.*, 2012) have recently been developed to automate the annotation and filtering of genomics variants. However, these systems are restrictive, as expansion and maintenance of annotation depends on the authors’ availability/willingness, and annotation and filtering are often combined, making integration with other tools challenging. Other approaches such as Bio2RDF (Belleau *et al.*, 2008) propose the conversion of annotation sources into Resource Description Framework (RDF) format that can be loaded into a triple store database for querying. This approach, although flexible because it allows independent integration of new annotation sources, presents scalability limitations and does not integrate well with existing command-line tools. Under production loads, the number of searches to annotate variants can become extremely large. For instance, the annotation of ∼30 million variants from 10 whole genome sequencing runs per day, with annotation extracted from 10 data sources would involve >300 million queries.

In this article, we present the Biological Reference Repository (BioR), a flexible and scalable infrastructure for the specific purpose of gene and variant annotation. BioR is built around a slightly modified version of the JSON format (http://www.json.org/), referred in this article as TJSON. To facilitate usability, BioR provides a toolkit (BioR toolkit) that includes a set of UNIX command-line functions to facilitate catalog management and annotation extraction. The BioR toolkit is engineered to work in high-performance computing environments and scale to multiple simultaneous instances.

## 2 METHODS AND RESULTS

### 2.1 The TJSON representation

The TJSON representation is used by catalogs and used as standard input/output for most of the functions of the BioR toolkit. The TJSON consists of a mix of tab-delimited values and JSON strings (see example below). Like JSON, TJSON is a compact, readable and hierarchical format that can be used to store one to many relationships present in relational annotation sources. TJSON was preferred over others like XML, as in addition to being readable, it is relatively compact. Like XML, it can represent complex hierarchical data structures into a single text string. The hierarchical structures existing in relational data sources are therefore maintained in BioR catalogs. JSON strings can easily be extracted from a TJSON and processed with JSON libraries in most programming languages like Perl, Java and Python. BioR provides commands necessary to retrieve nested values from JSON strings. An example of TJSON, where ‘\t’ is a tab character (typically non-displaying) acting as a column separator is here:

1024\t145.6\t{"_type":"gene","_strand":"+", "_minBP":10954,"_maxBP":11507,"note":"similarity to: 1 Protein", "GeneID":"100506145"}\t12.334

### 2.2 BioR toolkit

The BioR toolkit includes set of commands for the management of catalogs, extraction of annotation based on genomics coordinates, variant or gene information. These stand-alone commands that are executed like common UNIX commands leverage third-party JSON libraries to process JSON strings. TJSON is intentionally used as standard input/output by most of the BioR commands to enable the concatenation of multiple BioR commands into a single UNIX command using standard piping syntax. The user can add functions to the toolkit or operate on their data using conventional UNIX tools as long as the function operates on TJSON strings.

The BioR toolkit also includes commands to convert tab-delimited input file into TJSON strings (such as VCF and BED files) or convert TJSON into tab-delimited output file. Any metadata recorded in VCF or GFF style header (starting with ‘#’) in the input file will be carried through by the BioR toolkit functions to be recorded in the output file. The commands included in the BioR toolkit are listed in Supplementary Table S1.

Finally, the BioR toolkit supports two command-line utilities for annotating variants: (i) bior_snpeff, which integrates SnpEff annotations (Cingolani *et al.*, 2012), and (ii) bior_vep to annotate files using Ensemble’s variant effect predictor (www.ensembl.org/info/docs/variation/vep/).

### 2.3 BioR annotation catalogs

BioR catalogs are in a readable, indexable and schema-free format for storing and rapidly accessing arbitrary structured data such as genomic features, diseases, conditions, genetic tests and drugs. Catalogs are modular, based on specific data sources or tools, and can be built and queried independently of other catalogs. They use the TJSON representation to store annotation information and corresponding genomic coordinates. The first tab-delimited field is used to store the origin of the sequence (usually a chromosome). The next two fields record the start and end coordinates of a genomic interval for position-dependent annotations. These two fields are otherwise set to 0. These three fields are indexed by Tabix (Li, 2011). The last field is a JSON string that contains all the data from the original source.

To reduce storage footprint and accelerate coordinate-based searches, catalogs are compressed using the open source BGZip (Danecek *et al.*, 2011) and indexed using Tabix. The Tabix index file is stored in the same directory as the related catalog. BioR toolkit takes advantage of the Tabix library to perform coordinate-based overlap searches. BioR can also perform searches on identifiers that can be indexed using a BioR toolkit command for fast querying. Finally, to accelerate coordinate-based and variant-matching searches, a set of semantically consistent identifiers called Golden Identifiers are automatically indexed. These identifiers are implicitly used by some BioR commands (Supplementary Table S2).

### 2.4 Building BioR catalogs

The complexity of building BioR catalogs depends on the organization of data in the annotation source. Data available in tab-delimited text format can be readily converted to a BioR catalog using the command ‘bior_create_catalog’ and a configuration file describing each column. When annotations are extracted from complex systems such as relational databases, programming is required to reformat related tables into a single tab-delimited text. BioR catalogs must be created for each set of related tables the user wants to use.

### 2.5 BioR catalog library

BioR includes 19 documented catalogs built from the most commonly used data sources (Supplementary Table S3). It also includes a list of catalogs built from UCSC Genome Browser tracks (Kent *et al.*, 2002). To increase clinical applicability, pharmacogenomics catalogs built from PharmGKB, DrugBank and Therapeutic Target Database are also provided.

### 2.6 Example

The following example illustrates how sample variant rsIDs stored in the file rsID.txt can be annotated with European frequency from the 1000 Genomes Project. First, using the ‘bior_lookup’ command, rsIDs in the rsID.txt file are matched to entries in the dbSNP.tsv.bgz catalog containing the identifier ‘ID’. Matching entries in JSON format are piped to the function ‘bior_same_variant’. This function uses the Golden Identifiers present in the JSON string to look up allele frequencies in the KGenomes.tsv.gz catalog. Finally, the function ‘bior_drill’ and the Unix command ‘cut’ reformat the TJSON string into a tab-delimited output.

$ cat rsIDs.txt | bior_lookup -p ID –d dbSNP.tsv.bgz |

bior_same_variant -d KGenomes.tsv.gz |

bior_drill -c -1 -p INFO.EUR_AF | cut -f 1,3

This macro annotates 100 000 rsIDs in 2:23 min on a MacBook Pro 2.3 GHz Intel Core i7 with solid state drive and 8 G RAM.

## 3 RESULTS

BioR is an open annotation tool. It includes a toolkit with a base set of commands needed to build and index catalogs and retrieve annotations. Annotations can be retrieved based on location (genomic coordinates) or identifiers. The TJSON format is used for catalogs and as input/output for most of the toolkit functions facilitating the assembly of complex pipelines. Because the TJSON format is readable, users can design their own scripts to extract annotation from catalogs. Scripts can also be intermixed with toolkit commands as long as the TSJON format is maintained. This stream-based approach on which BioR is based significantly reduces memory footprint. In addition, the BioR toolkit is inherently parallel and can be configured to take advantage of computers with multi-core architectures. BioR catalogs can easily be combined into new catalogs to decrease retrieval time by avoiding multiple cross-catalog queries. In conclusion, BioR is a rapid and flexible system for annotating high-throughput genomics experiments.

## Supplementary Material

Supplementary Data
