# Supplementary material for: The Biological Reference Repository (BioR): a rapid and flexible system for genomics annotation
Source: Bioinformatics. 2014 Mar 10;30(13):1920–2. doi: 10.1093/bioinformatics/btu137 (PMC4071205; doi:10.1093/bioinformatics/btu137)
Supplement: Supplementary Data [file supp_30_13_1920__index.html]

The Biological Reference Repository (BioR): a Rapid and Flexible System for Genomics Annotation — The Biological Reference Repository (BioR): a rapid and flexible system for genomics annotation — The Biological Reference Repository (BioR): a rapid and flexible system for genomics annotation — Supplementary Data 

# The Biological Reference Repository (BioR): a rapid and flexible system for genomics annotation

## Supplementary Data

files

**Files in this Data Supplement:**

- Supplementary Data - docx file
- Supplementary Data - docx file
