# Supplementary material for: The Biological Reference Repository (BioR): a rapid and flexible system for genomics annotation
Source: Bioinformatics. 2014 Mar 10;30(13):1920–2. doi: 10.1093/bioinformatics/btu137 (PMC4071205; doi:10.1093/bioinformatics/btu137)
Supplement: Supplementary Data [file supp_btu137_BioRQuickstart2.docx]

The Biological Repository (BioR) Quick-start

This is a quick start for those in a hurry.  For more detailed instructions, please see the user manual on the following page: <http://bioinformaticstools.mayo.edu/research/bior/>

BioR tools and their uses (shortlist)

| **Tool Command** | **Input Format** | **Output Format** | **Function** |
| --- | --- | --- | --- |
| bior_overlap | TJSON | TJSON | Extract from catalog all the attributes based on genomic coordinates |
| bior_same_variant | TJSON | TJSON | Matches variants based on position, reference, and alternate alleles |
| bior_lookup | TJSON | TJSON | Matches based on a string/identifier |
| bior_drill | TJSON | Tab-Delim | Extracts key-value relationships from JSON object |
| bior_pretty_print | TJSON | JSON | Prints the JSON to the screen in a more readable way |
| bior_vcf_to_tjson | VCF | TJSON | Converts VCF format to JSON objects |
| bior_bed_to_json | BED | TJSON | Converts BED format to JSON objects |
| bior_snpeff | VCF | TJSON | Wraps the SNPEFF tool (if installed) |
| bior_vep | VCF | TJSON | Wraps the VEP tool (if installed) |
| bior_annotate | VCF | TJSON | Wrapper to get commonly used variant annotations |
| bior_index_catalog | TJSON | INDEX | Creates and index on a string/identifier |

## Prerequisites:

1) Make sure that you have BioR and its dependencies installed correctly and in your path.  This includes Java JDK 1.7, Tabix and BGZIP.  Most BioR functions will still work if you don’t install SNPEFF/VEP and all of their dependencies, but bior_annotate will have limited functionality and bior_vep and bior_snpeff will not work. The environment variable, $BIOR_LITE_HOME represents the location where BioR is installed.

2) To check it is installed and on your path, execute bior_pretty_print –h at the command line: it should output a description and the text on how to use bior_pretty_print.

**$ bior_pretty_print –h**

NAME

bior_pretty_print -- prints out a single data row in a readable format

SYNOPSIS

bior_pretty_print [--row-number <number>] [--log] [--help]

DESCRIPTION

…

3) Ready to run examples are available in $BIOR_LITE_HOME/examples.

## Example 1:

Change directory to $BIOR_LITE_HOME/examples/quickstart1. The first example in this guide is to convert gene names into gene positions, which follows this process:

1. Get the gene list
2. Find a catalog that has a gene name in it
   - in this example: $BIOR_LITE_HOME/examples/quickstart1/data/NCBIGene/GRCh37_p10/genes.tsv.bgz
3. Look up your gene name in that catalog with bior_lookup
4. Extract the key/value pairs that you are interested with bior_drill

**STEP1**. The file geneList.txt contains a list of genes. Cat the file to see what you are starting with.

**$ cat geneList.txt**

BRCA1

BRCA2

CD28

ZBTB7A

**$**

**STEP2**. Look at the contents of the gene catalog to see what fields are there (NOTE: use ***gzcat*** on a mac or ***zcat*** on Linux). For brevity, we are only showing selected portions in this manual, but the complete outputs are given in STEP1.txt, STEP2.txt, etc.

**$ gzcat data/NCBIGene/GRCh37_p10/genes.tsv.bgz | bior_pretty_print**

… {

"_landmark": "1",

"_strand": "+",

"_minBP": 10954,

"_maxBP": 11507,

"gene": "LOC100506145"

…

}

**$**

**STEP3**. Find the genes in geneList.txt in the gene field of the genes.tsv.bgz catalog using bior_lookup:

**$ cat geneList.txt | bior_lookup -p gene -d data/NCBIGene/GRCh37_p10/genes.tsv.bgz | bior_pretty_print**

# COLUMN NAME COLUMN VALUE

- ----------- ------------

1 UNKNOWN_1 BRCA1

2 bior.gene37p10 {

…

"_landmark": "17",

"_strand": "-",

"_minBP": 41196312,

"_maxBP": 41277500,

"gene": "BRCA1",

…

"MIM": "113705"

}

**$**

**STEP4**. Create a simple tab-delimited file of the gene and position information using bior_drill:

**$ cat geneList.txt | bior_lookup -p gene -d data/NCBIGene/GRCh37_p10/genes.tsv.bgz | bior_drill -p _landmark -p _strand -p _minBP -p _maxBP > genePos.txt**

**$ cat genePos.txt**

…

#UNKNOWN_1 bior.gene37p10._landmark bior.gene37p10._strand bior.gene37p10._minBP bior.gene37p10._maxBP

BRCA1 17 - 41196312 41277500

BRCA2 13 + 32889617 32973809

CD28 2 + 204571198 204603635

ZBTB7A 19 - 4045216 4066816

**$**

## Example 2:

The second example shows briefly how to annotate a VCF file. In this example, we are interested in finding out if any of my variants are clinically relevant from a subset of the ClinVar database (http://www.ncbi.nlm.nih.gov/variation/docs/human_variation_vcf/).  From the glossary of the catalog source ([http://www.ncbi.nlm.nih.gov/variation/docs/glossary](http://www.ncbi.nlm.nih.gov/variation/docs/glossary/)), CLNSIG is the annotation we are looking for, since it classifies variants as pathogenic, non-pathogenic, drug-response, etc. For this example, you will need to change directory to $BIOR_LITE_HOME/examples/quickstart2. The file example.vcf contains a VCF file of genetic variants.

To annotate the VCF:

1. VCFs must be converted into a tjson file before searching using bior_vcf_to_tjson
2. We need to use bior_same_variant to match alternate alleles between our VCF and the ClinVar catalog
3. Drill out the key/value pairs of interest
4. Convert back to VCF for future use or pipe directly into another command

**$ cat example.vcf | bior_vcf_to_tjson | bior_same_variant -d data/dbSNP/137/clinvarPart.tsv.bgz | bior_drill -p INFO.CLNSIG | bior_tjson_to_vcf**

##fileformat=VCFv4.0

##INFO=<ID=bior.clinvar137.INFO.CLNSIG,Number=.,Type=String,Description="VCF INFO. A string that describes the variant's clinical significance, where 0 - unknown, 1 - untested, 2 - non-pathogenic, 3 - probable-non-pathogenic, 4 - probable-pathogenic, 5 - pathogenic, 6 - drug-response, 7 - histocompatibility, 255 - other.">

#CHROM POS ID REF ALT QUAL FILTER INFO

13 32907403 . T C . . bior.clinvar137.INFO.CLNSIG=1

15 3240324 . N C . . .

**$**

 *Notice the “INFO.CLINSIG” instead of just “CLINSIG”. This is because the CLINSIG key is in the hierarchy of INFO {} (see STEP5.txt).
