# Supplementary material for: The Biological Reference Repository (BioR): a rapid and flexible system for genomics annotation
Source: Bioinformatics. 2014 Mar 10;30(13):1920–2. doi: 10.1093/bioinformatics/btu137 (PMC4071205; doi:10.1093/bioinformatics/btu137)
Supplement: Supplementary Data [file supp_btu137_BioR_Supplementary_Material2.docx]

# Supplementary Materials

| Command | Input, Output | Description |
| --- | --- | --- |
| **Transform Functions** |  |  |
| bior_overlap | TJSON, TJSON | Extract annotations from a catalog based on genomic location overlap. The overlap is computed from the Start and End genomics position of a variant. |
| bior_same_variant | TJSON, TJSON | Extract annotations from a catalog based on variant position, reference and alternate allele definition. |
| bior_lookup | TJSON, TJSON | Extract annotations from a catalog based on matching values of an identifier. |
| bior_snpeff | TJSON, TJSON | Use SNPEffect^1^ to annotate variants. Chromosome ID, Start and Stop genomics position, reference and alternate allele of the variant is required . |
| bior_vep | TJSON, TJSON | Use VEP^2^ to annotate variants. Chromosome ID, Start and Stop genomics position, reference and alternate allele of the variant is required. |
| bior_drill | TJSON, TJSON | Extract an element from nested JSON string. |
| bior_compress | TJSON, TJSON | Compress entries from provided set of identifiers into a single entry with each value separated by a delimiter. |
| **Utility Functions** |  |  |
| bior_index_catalog | identifier, index | Index the specified identifier in a catalog. Indices a stored in a separate index file. |
| bior_create_catalog | TJSON, catalog | Convert a text tabulated file into a catalog. Chromosome ID, Start and End genomics position fields have to be explicitly named. |
| bior_ create_catalog_props | catalog, property | Create property files from the metadata extracted from a catalog. Property files are needs for proper metadata handling. |
| bior_create_config_for_tab_to_tjson | TSV, config_file | Create a configuration file that describes column description. This file is needed when uploading a tab delimited file. |
| **Input/Output Functions** |  |  |
| bior_vcf_to_tjson | VCF, TJSON | Load a VCF file and convert to TJSON format. |
| bior_tjson_to_vcf | TJSON, VCF | Convert TJSON to VCF format for file output. |
| bior_bed_to_tjson | BED, TJSON | Load a BED file and convert to TJSON format. |
| bior_tab_to_tjson | TSV, TJSON | Load a tab-delimited file and convert to TJSON format. |
| bior_pretty_print | TJSON, STDOUT | Convert TJSON in a readable format for screen or file output. |
| **Miscellaneous Functions** |  |  |
| bior_annotate | VCF, TJSON | Append to the VCF ‘info’ field a set of commonly used annotations. |

Table S1: List of commands available in the BioR Toolkit. Detailed description and example is displayed when executing the command with the –h flag.
^1^Cingolani, P. et al. (2012) A program for annotating and predicting the effects of single nucleotide polymorphisms, SnpEff: SNPs in the genome of Drosophila melanogaster strain w1118; iso-2; iso-3. Fly (Austin). 6(2) :p. 80-92.
^2^McLaren W et al. (2010) Deriving the consequences of genomic variants with the Ensembl API and SNP Effect Predictor. BMC Bioinformatics 26(16):2069-70

| ‘Golden Identifier’ | Used by | Definition |
| --- | --- | --- |
| _landmark | bior_overlap, bior_same_variant | Chromosome, or sequence ID where the interval is located |
| _minBP | bior_overlap, bior_same_variant | Minimum 1-based position (e.g. NCBI coordinates) on the landmark sequence |
| _maxBP | bior_overlap, bior_same_variant | Maximum 1-based position on the landmark sequence |
| _refAllele | bior_same_variant | REF as in VCF standard |
| _altAlleles | bior_same_variant | ALT as in VCF standard |

Table S2: List of indexed ‘golden identifiers’ that are used to accelerate coordinate-base searches and variant matching

| Datasource | URL | Version |
| --- | --- | --- |
| 1000Genomes | <http://www.1000genomes.org/category/ftp> | 20110521 |
| BGI | http://soap.genomics.org.cn/soapsnp.html | hg19 |
| COSMIC | http://cancer.sanger.ac.uk/cancergenome/projects/cosmic/ | V63 |
| dbSNP | http://www.ncbi.nlm.nih.gov/snp/ | 137 |
| ESP6500 | https://esp.gs.washington.edu/drupal/ | build37 |
| HapMap | http://hapmap.ncbi.nlm.nih.gov | 2010-08_phaseII+III |
| HGNC | http://www.genenames.org | 2012_08_12 |
| miRBase | http://www.mirbase.org | 8_12_12 |
| NCBIGene | http://www.ncbi.nlm.nih.gov/gene | GRCh37_p10 |
| OMIM | http://www.omim.org | 2013_02_27 |
| PharmGKB | http://www.[pharmgkb.org/downloads/](http://www.pharmgkb.org/downloads/) | June 2013 |
| DrugBank | http://www.drugbank.ca/downloads | 3.0 |
| Therapeutic Target Database | http://bidd.nus.edu.sg/group/cjttd/TTD_Download.asp | 4.3.02 |
| UCSC | http://hgdownload.cse.ucsc.edu/goldenPath/hg19/database/  (note catalogs were created for each UCSC track) | hg19 |

Table S3: list of data sources from which BioR catalogs are derived. A description of the catalog is available at [http://bioinformaticstools.mayo.edu](http://bioinformaticstools.mayo.edu/)
